# The Contribution of L-Type Ca_v_1.3 Channels to Retinal Light Responses

**DOI:** 10.3389/fnmol.2017.00394

**Published:** 2017-12-05

**Authors:** Liheng Shi, Janet Ya-An Chang, Fei Yu, Michael L. Ko, Gladys Y.-P. Ko

**Affiliations:** ^1^Department of Veterinary Integrative Biosciences, Texas A&M University, College Station, TX, United States; ^2^Texas A&M Institute of Neuroscience, Texas A&M University, College Station, TX, United States

**Keywords:** L-type voltage-gated calcium channel, photoreceptor, ribbon synapses, electroretinogram

## Abstract

L-type voltage-gated calcium channels (LTCCs) regulate tonic neurotransmitter release from sensory neurons including retinal photoreceptors. There are three types of LTCCs (Ca_v_1.2, Ca_v_1.3, and Ca_v_1.4) expressed in the retina. While Ca_v_1.2 is expressed in all retinal cells including the Müller glia and neurons, Ca_v_1.3 and Ca_v_1.4 are expressed in the retinal neurons with Ca_v_1.4 exclusively expressed in the photoreceptor synaptic terminals. Mutations in the gene encoding Ca_v_1.4 cause incomplete X-linked congenital stationary night blindness in humans. Even though Ca_v_1.3 is present in the photoreceptor inner segments and the synaptic terminals in various vertebrate species, its role in vision is unclear, since genetic alterations in Ca_v_1.3 are not associated with severe vision impairment in humans or in Ca_v_1.3-null (Ca_v_1.3^−/−^) mice. However, a failure to regulate Ca_v_1.3 was found in a mouse model of Usher syndrome, the most common cause of combined deafness and blindness in humans, indicating that Ca_v_1.3 may contribute to retinal function. In this report, we combined physiological and morphological data to demonstrate the role of Ca_v_1.3 in retinal physiology and function that has been undervalued thus far. Through *ex vivo* and *in vivo* electroretinogram (ERG) recordings and immunohistochemical staining, we found that Ca_v_1.3 plays a role in retinal light responses and synaptic plasticity. Pharmacological inhibition of Ca_v_1.3 decreased *ex vivo* ERG a- and b-wave amplitudes. In Ca_v_1.3^−/−^ mice, their dark-adapted ERG a-, b-wave, and oscillatory potential amplitudes were significantly dampened, and implicit times were delayed compared to the wild type (WT). Furthermore, the density of ribbon synapses was reduced in the outer plexiform layer of Ca_v_1.3^−/−^ mice retinas. Hence, Ca_v_1.3 plays a more prominent role in retinal physiology and function than previously reported.

## Introduction

L-type voltage-gated calcium channels (LTCCs) are multi-subunit channel complexes composed of a pore-forming α1 subunit and auxiliary β and α2δ subunits. In the retina and inner ear, LTCCs mediate tonic neurotransmitter release from the ribbon synapses (Barnes and Kelly, 2002; Catterall et al., 2005; Dolphin, 2006). In cochlea hair cells, calcium influx through LTCCs triggers glutamate release from the inner hair cells onto the spiral ganglion neurons and participates in the fine-tuning of frequency detection of sound (Sheets et al., 2012; Joiner and Lee, 2015). In the retina, photoreceptors and bipolar cells release glutamate continuously in the dark as a result of depolarization-evoked activation of LTCCs (Barnes and Kelly, 2002). In addition to neurotransmitter release, LTCCs are involved in the regulation of membrane excitability, resonance properties, endocytosis, and synaptic plasticity at reciprocal synapses in the photoreceptors, bipolar cells, and amacrine cells (Palmer et al., 2003a,b; Hull and von Gersdorff, 2004; Vigh et al., 2005; Hull et al., 2006a). Thus, LTCCs may participate in multiple functions in the retina. There are three types of LTCCα1 subunits, Ca_v_1.2, Ca_v_1.3, and Ca_v_1.4, present in the retina (Morgans, 2001; Barnes and Kelly, 2002; Ko et al., 2007; Lee et al., 2015). Among them, Ca_v_1.4 is strongly expressed at the ribbon synapses (Morgans, 2001; Lee et al., 2015), and its function is the most well-characterized in the retina, since mutations in Ca_v_1.4 cause X-linked incomplete congenital stationary night blindness type 2 (CSNB2) in humans (Bech-Hansen et al., 1998; Liu et al., 2013). This is due to the essential role of Ca_v_1.4 in the formation of ribbon synapses between photoreceptor terminals and the second-order neurons during development (Liu et al., 2013). However, there are small residual inner retinal light responses recorded from CSNB2 patients (Miyake et al., 1986; Bradshaw et al., 2004) suggesting that there might be other LTCCα1 subunits present at the photoreceptor synaptic terminals to transmit light information to the inner retina.

Compared to Ca_v_1.4, the functional roles of Ca_v_1.2 and Ca_v_1.3 are less known in the retina. While Cav1.2 is expressed in all retinal cells including the Müller glia, Cav1.3 is expressed only in the retinal neurons (Xu et al., 2002; Ko et al., 2007). Mutations of Ca_v_1.2 or Ca_v_1.3 cause severe cardiovascular dysfunctions in humans and animals (Seisenberger et al., 2000; Splawski et al., 2004; Striessnig et al., 2014; Pinggera et al., 2015; Pinggera and Striessnig, 2016), and dysregulation of Ca_v_1.3 severely impairs hearing (Platzer et al., 2000; Seisenberger et al., 2000). In Ca_v_1.3-null mutant (Ca_v_1.3^−/−^) mice, the retina displays mild morphological changes in the outer plexiform layer (OPL) where photoreceptors and secondary neurons form synaptic contacts (Busquet et al., 2010). Even though there is no specific report on vision loss in humans or animals with Ca_v_1.3 mutations, a failure to regulate Ca_v_1.3 is found in a mouse model of Usher syndrome, the most common cause of combined deafness and blindness in humans (Petit, 2001; Kersten et al., 2010; Joiner and Lee, 2015), indicating that Ca_v_1.3 may contribute to retinal function and physiology that requires further investigation.

One potential function of Ca_v_1.3 in the retina may be in adaptive processes to external stimulation. In cultured retinal amacrine and ganglion cells, activation of glutamate receptors causes a rapid internalization of Ca_v_1.3 but not Ca_v_1.2 (Mizuno et al., 2010) suggesting that Ca_v_1.3 is more “plastic” and responsive to potential light/dark stimulations. The plasticity of Ca_v_1.3 may serve as an acute adaptation to protect the inner retinal circuitry against glutamate excitotoxicity (Mizuno et al., 2010). The mRNA and protein expressions of Ca_v_1.3 are rhythmic in circadian oscillations, in which there are more Ca_v_1.3 subunits inserted in the plasma membrane with larger Ca_v_1.3-currents recorded at night than during the day in the avian retina (Ko et al., 2007; Ko M. L. et al., 2009). This indicates that Ca_v_1.3 may participate in fine-tuning the retinal light responses to anticipate the ambient light changes throughout the course of a day (Ko et al., 2007; Ko G. Y. et al., 2009; Ko M. L. et al., 2009). To further understand the role of Ca_v_1.3 in mammalian retinal physiology and function, we employed *ex vivo* and *in vivo* ERG studies to decipher the contribution of Ca_v_1.3 to retinal light responses. Using *ex vivo* ERG recordings, we were able to isolate the a-wave from the b-wave and carefully analyze the contribution of Ca_v_1.3 in the outer (a-wave) and inner (b-wave) retina. We further compared the retinal light responses from both outer and inner retina among the wild type (WT), Ca_v_1.3 heterozygous mutant (Ca_v_1.3^+/−^), and Ca_v_1.3^−/−^ mice. Combined with our morphological observations, we provide new evidence on the contribution of Ca_v_1.3 to retinal light responses and synaptic transmission.

## Materials and methods

### Animals

Male C57BL/6J mice (WT) were purchased from Harlan (Houston, TX, USA) and used at 2–3 months old in this study. The Ca_v_1.3^−/−^ mice (C57BL/6J background) were originally developed by Dr. Jörg Striessnig (University of Innsbruck, Innrain, Innsbruck, Austria; Platzer et al., 2000). The Ca_v_1.3^+/−^ (heterozygous) breeding pair for generating Ca_v_1.3^−/−^ (homozygous knockout) was from Dr. Amy Lee (University of Iowa, Iowa City, IA, USA). The Ca_v_1.3^−/−^, Ca_v_1.3 ^+/−^, and Ca_v_1.3^+/+^ (WT) littermates used in this study were produced at Texas A&M University (College Station, TX, USA). All animal experiments were approved by the Institutional Animal Care and Use Committee of Texas A&M University. Mice were housed under temperature and humidity-controlled conditions with 12:12 h light-dark cycles.

### HEK cell culture and transfection

The human HEK 293 cell line was purchased from American Type Culture Collection (ATCC, Manassas, VA, USA). The cells were maintained in DMEM (BioWhittaker, Walkersville, MD, USA) containing 10% FBS (HyClone, Pittsburgh, PA, USA), 50 u/ml penicillin/50 μg/ml streptomycin (Sigma-Aldrich, St. Louis, MO, USA), 1 mM sodium pyruvate (Life Technologies, Carlsbad, CA, USA), and 1x non-essential amino acids (Life Technologies) at 37°C under 5% CO_2_. Cells were cultured on coverslips and placed in a 24-well culture plate. Cells were seeded in each well to 70–80% confluence 24 h prior to the transfection. Transfections were performed using Lipofectamine 2000 Transfection Reagent (Life Technologies) according to the manufacturer's protocol. The calcium channel α2δ1 subunit (rat) expression vector was a gift from Dr. Terrance P. Snutch (University of British Columbia, Vancouver, Canada). The pCDNA-Ca_v_1.2 α1 subunit was originally generated by Dr. Diane Lipscombe (Brown University, Providence, Rhode Island, USA) and distributed through Addgene (Cambridge, MA, USA). The pCDNA-Ca_v_1.3 α1 subunit (mouse) was from Dr. Amy Lee. The pCMV-Sport-β2 subunit (mouse) was purchased from MGC cDNA clones collection (Dharmacon, GE, Lafayette, CO). Up to 500 ng DNA (150 ng for each plasmid) was transfected into the cultured HEK cells, and culture media was exchanged 12 h after transfection. Electrophysiological recordings were carried out 60 h after the transfection.

### Patch-clamp electrophysiological recordings

The whole cell patch-clamp recordings for LTCCs were carried out as previously described (Ko et al., 2007; Shi et al., 2009). The external solution was (in mM): 145 TEACl, 9 BaCl_2_, 0.5 MgCl_2_, 5.5 glucose, 0.1 NiCl_2_, and 5 HEPES, pH 7.4 adjusted with TEAOH. The pipette solution was (in mM): 125 Cs acetate, 20 CsCl, 3 MgCl_2_, 10 EGTA, and 5 HEPES, pH adjusted with CsOH. The holding potential for transfected HEK cells was set at −65 mV. Currents were recorded at room temperature using an A-M Systems model 2400 patch-clamp amplifier (Sequim, WA, USA). Signals were low-pass filtered at 2 kHz and digitized at 5 kHz with Digidata 1550A interface and pCLAMP 10.5 software (Molecular Devices, Sunnyvale, CA, USA). The Ba^2+^ current was recorded immediately after the whole-cell configuration was formed by gentle suction, and the ramp-voltage command (−80 to +60 mV in 500 ms) was applied to elicit Ba^2+^ currents. D-cis-diltiazem (diltiazem; Sigma-Aldrich) was first dissolved in water and further diluted in external recording solutions to the appropriate final concentrations as denoted in the results. The cells were first recorded in normal external solution for baseline currents, followed by perfusion with diltiazem (DIL), and the ramp-voltage command was elicited once per minute after perfusion with DIL. The controls were recorded following the same protocol but perfused with an external solution without DIL. The peak current amplitudes were normalized as the percentage (%) to the original baseline amplitude (set at 100%) for each cell recorded.

### *Ex vivo* electroretinogram (ERG) recordings

Mice were dark adapted for at least 3 h prior to the recordings. All experiments were performed under dim red light as previously described (Kolesnikov and Kefalov, 2012). This *ex vivo* ERG recording technique and the configuration of the recording chamber were originally designed by Dr. Vladimir Kefalov (Washington University, St. Louis, MO, USA). Mouse retinas were dissected out in oxygen saturated dissection medium containing 1 mg/ml BSA and 13.6 mg/ml L-15 (Sigma-Aldrich) at 37°C. Retinas were transferred to an *ex vivo* ERG recording chamber (OcuScience, Henderson, NV, USA) and perfused with a buffer containing (in mM): 112 NaCl, 3.6 KCl, 2.4 MgCl_2_, 20 NaHCO_3_, 3 Na succinate, 0.02 EDTA, 10 Glucose, 10 HEPES (pH 7.4), 0.72 mg/ml L-15, 0.1% MEM vitamins, and MEM non-essential amino acids (Sigma-Aldrich) at 37°C. The electrode solution in the recording chamber contained (in mM): 140 NaCl, 2.4 MgCl_2_, 1.2 CaCl_2_, 3 HEPES (pH 7.4). In order to isolate the ERG a-wave and observe photoreceptor responses, the perfusion solution was supplemented with 2 mM L-glutamate and 10 μM DL-AP-4, and the electrode solution was supplemented with 2 mM L-glutamate and 10 mM BaCl_2_ to block the higher order photo-responses (Kolesnikov and Kefalov, 2012)_._ Nitrendipine (EMD Millipore, Billerica, MA) was first dissolved in dimethylsulfoxide (DMSO), and further diluted in the external recording solution to 10 μM as the final concentration. A portable ERG device (OcuScience) was used for *ex vivo* ERG recordings. The ERG measurements were carried out sequentially at light intensities of 0.1, 0.3, 1.0, and 3.0 cd·s/m^2^. Each ERG response was an average of 4 light flashes at a specific light intensity. A 1-min recovery period was allowed between different intensities. The amplitudes and implicit times of a- and b-waves were recorded and analyzed by using the ERGView 4.4 software (OcuScience). *Ex vivo* results were normalized to the original baseline amplitude prior to the perfusion with the calcium channel inhibitors (diltiazem or nitrendipine) and reported as a change in percentage from the original baseline amplitude.

### *In vivo* ERG recordings

Male mice at 3 months old were used for *in vivo* ERG recordings (performed as previously described; Chang et al., 2015). Mice were dark-adapted for at least 8 h then anesthetized with an intraperitoneal injection of Avertin (0.5 mL/25 g-body weight of 2% 2,2,2-tribromoethanol, 1.25% *tert*-amyl alcohol; Fisher Scientific, Pittsburgh, PA, USA). Pupils were dilated using a single drop of a 1% tropicamide and 2.5% phenylephrine mixture for 5 min. Mice were placed on a heating pad to maintain body temperatures at 37°C. The ground electrode was placed on the tail and the reference electrode placed under the skin in the cheek below the eye. A drop of Goniovisc (Hub Pharmaceuticals, Rancho Cucamonga, CA, USA) was applied on the surface of the cornea to keep it moist, and the threaded recording electrode conjugated to a mini contact lens (Ocuscience) was placed on top of the cornea. A dim red light was used for all preparatory procedures but was turned off during the ERG recording. A portable ERG device was used for measurements of light responses from a series of light stimulations at 0.1, 0.3, 1, 3, 10, and 25 cd·s/m^2^. Low light intensities (0.1, 0.3, 1.0, and 3.0 cd·s/m^2^) were flashed four times at 10 s intervals and the traces were averaged for a final ERG measurement. High light intensities (10 and 25 cd·s/m^2^) only had one flash. A 1 min recovery period was programmed between each light intensity. Amplitudes and implicit times of a- and b-waves were recorded and analyzed, and the oscillatory potentials were band-pass filtered between 100 and 300 Hz using the ERGView 4.4 software. The a-wave implicit time was measured from the onset of the light stimulus to the most hyperpolarized point (trough) of the a-wave, while the b-wave implicit time was measured from the onset of the light stimulus to the highest peak of the b-wave.

### Immunohistochemistry

Ca_v_1.3^−/−^ and Ca_v_1.3^+/+^ (WT) littermate mice were first anesthetized with isoflurane followed by cervical dislocation. The eyes were excised and fixed in Zamboni fixative (American Matertech Scientific Inc, Lodi, CA, USA) then cryo-protected in a 30% sucrose-PBS solution. Ca_v_1.3^−/−^ and Ca_v_1.3^+/+^ eyes were embedded side by side in Tissue-Tek O.C.T. Compound (Sakura Finetek Inc, Torrance, CA, USA) and stored at −80°C. The frozen eye sections (10 μm) were cut using a cryostat (Leica Biosystem, Buffalo Grove, IL, USA) and mounted on glass slides. The sections were washed with PBS and incubated with a blocking solution containing 10% goat serum for 2 h at room temperature, followed by incubation with the primary antibodies (anti-Ribeye+anti-Ca_v_1.4 or anti-Ca_v_1.3 alone) at 4°C overnight. Sections were then washed three times with PBS containing 0.1% Triton 100 (PBST), incubated with a secondary antibody at room temperature for 2 h in a dark chamber, washed with PBST, and covered with the ProLong Gold antifade reagents containing 4′,6-diamidino-2-phenylindole (DAPI; Life Technologies) and a glass coverslip. The primary antibodies used were rabbit anti-Ca_v_1.3 (1:100; Chemicon/Millipore Sigma, St. Louis, MO, USA), mouse anti-ribeye (1:100; Millipore) and rabbit anti-Ca_v_1.4 (1:1000; a generous gift from Dr. Amy Lee, University of Iowa, Iowa City, IA). The secondary antibodies used were Alexa fluor 488 goat anti-rabbit IgG (1:200; Life Technologies) and Cy5 goat anti-mouse IgG (1:200; Abcam, Cambridge, MA, USA). The images were captured with a Zeiss LSM 780 NLO Multiphoton Microscope (Carl Zeiss AG, Oberkochen, Germany). The images from WT or Ca_v_1.3^−/−^ were taken under the identical setting (light intensity, magnification, and capture time) for Ribeye or Ca_v_1.4.

Quantification of Ribeye positive and Ca_v_1.4 positive synaptic terminals: After images were captured, we used “Fiji,” an image processing package that is an open-source platform for biological image analysis (Schindelin et al., 2012) to analyze our images. The “analyze particle” function in the Image J (software in “Fiji”) to quantify all fluorescent positive structures (as in pixels). Briefly, the threshold of individual fluorescent channel (red for Ribeye positive and green for Ca_v_1.4 positive) was automatically adjusted. The setting in the Image J software: particle size was from 0 to infinity, and circularity was from 0 to 1.0. We also used the “Coloc 2” (available at https://imagej.net/Coloc_2), a plugin utility to determine the colocalization of Ribeye and Ca_v_1.4. One stained retinal section was used per mouse. Three areas per retinal section were randomly selected. Channel 1 was assigned as red fluorescence (Ribeye positive), and channel 2 was assigned as green fluorescence (Ca_v_1.4 positive). The Li's Intensity Correlation Quotient (ICQ) value (Li et al., 2004) was generated to determine the degree of Ribeye and Ca_v_1.4 colocalization: for colocalized staining 0 < ICQ ≤ 0.5; ICQ = ~0 for random staining; for segregated staining 0 > ICQ ≥ −0.5.

### Western blotting

Samples were collected and prepared as described previously (Ko M. L. et al., 2009; Huang et al., 2013; Lin et al., 2015). Retinas were homogenized in a Tris lysis solution (50 mM Tris, 1 mM EDTA, 150 mM NaCl, 1% NP-40) including phosphatase (50 mM NaF, 1 mM Na_3_VO_4_) and protease inhibitors (Sigma-Aldrich). After centrifugation to remove cellular debris, an equal volume of 2x Laemmli buffer was added to each sample lysate, then the samples were heated at 95°C for 5 min. Proteins were separated by SDS-PAGE (10% gels) for 1–2 h. Proteins were then transferred to nitrocellulose membranes and probed by primary antibodies. The primary antibodies used were rabbit anti-Ca_v_1.3 antibody (1:1,000; Chemicon/ Millipore Sigma) and rabbit anti-actin antibody (1:1,000; Cell Signaling Technology, Danvers, MA, USA). Actin was used for loading controls. The secondary antibody (goat anti-rabbit) conjugated to horseradish peroxidase (1:1,000; Cell Signaling Technology) and the Femto and Pico electrochemiluminescense (ECL) kits (Pierce ThermoFisher Scientific, Waltham, MA, USA) were used to visualize the blots.

### Statistical analyses

All data are presented as mean ± SEM (standard error of mean). Based on this study, as well as previously published data by us and our unpublished results, the data for our patch-clamp electrophysiological recordings, *in vivo* ERG, *ex vivo* ERG, and morphological analyses were all in normal distribution. One-way analysis of variance (ANOVA) with Tukey *post hoc* tests were used for statistical analyses between the various treatment groups or between the WT, Ca_v_1.3^+/−^ (heterozygous mutant), and Ca_v_1.3^−/−^ homozygous mutant groups. The Student's *t*-test was used to compare between WT and Ca_v_1.3 ^−/−^. Throughout, *p* < 0.05 was regarded as significant.

## Results

### Distinguishing Ca_v_1.2 currents from Ca_v_1.3 through pharmacological inhibition

We first determined which pharmacological inhibitor could effectively differentiate Ca_v_1.2 from Ca_v_1.3, since there is no commercially available inhibitor that will selectively block Ca_v_1.2 but not Ca_v_1.3 (Cooper et al., 1987; Xu and Lipscombe, 2001). Among the LTCC inhibitors, dihydropyridines, phenylalkylamines, and benzothiazepines have lower affinities for Ca_v_1.3 compared to Ca_v_1.2 (Cai et al., 1997; Hockerman et al., 2000; Schnee and Ricci, 2003; Baumann et al., 2004; Tarabova et al., 2007; Bissig et al., 2013; Berkowitz et al., 2014). Particularly, the half maximal inhibitory concentration (IC50) of diltiazem (DIL) for Ca_v_1.3 is more than 10 times higher than Ca_v_1.2 (Cai et al., 1997; Hockerman et al., 2000; Schnee and Ricci, 2003; Baumann et al., 2004; Tarabova et al., 2007; Bissig et al., 2013; Berkowitz et al., 2014), so we took advantage of using DIL at a lower concentration to inhibit Ca_v_1.2 with minimal effects on Ca_v_1.3. However, a caveat we faced was that the effectiveness of DIL on Ca_v_1.2 vs. Ca_v_1.3 was not compared in the same cell type or preparation in previous reports (Cai et al., 1997; Hockerman et al., 2000; Schnee and Ricci, 2003; Baumann et al., 2004; Tarabova et al., 2007; Bissig et al., 2013; Berkowitz et al., 2014). Hence, we first set forth using HEK 293 cells transfected with Ca_v_1.2 or Ca_v_1.3 and recorded the LTCC currents to identify a concentration of DIL that would inhibit Ca_v_1.2 without affecting Ca_v_1.3.

**Figure 1:**
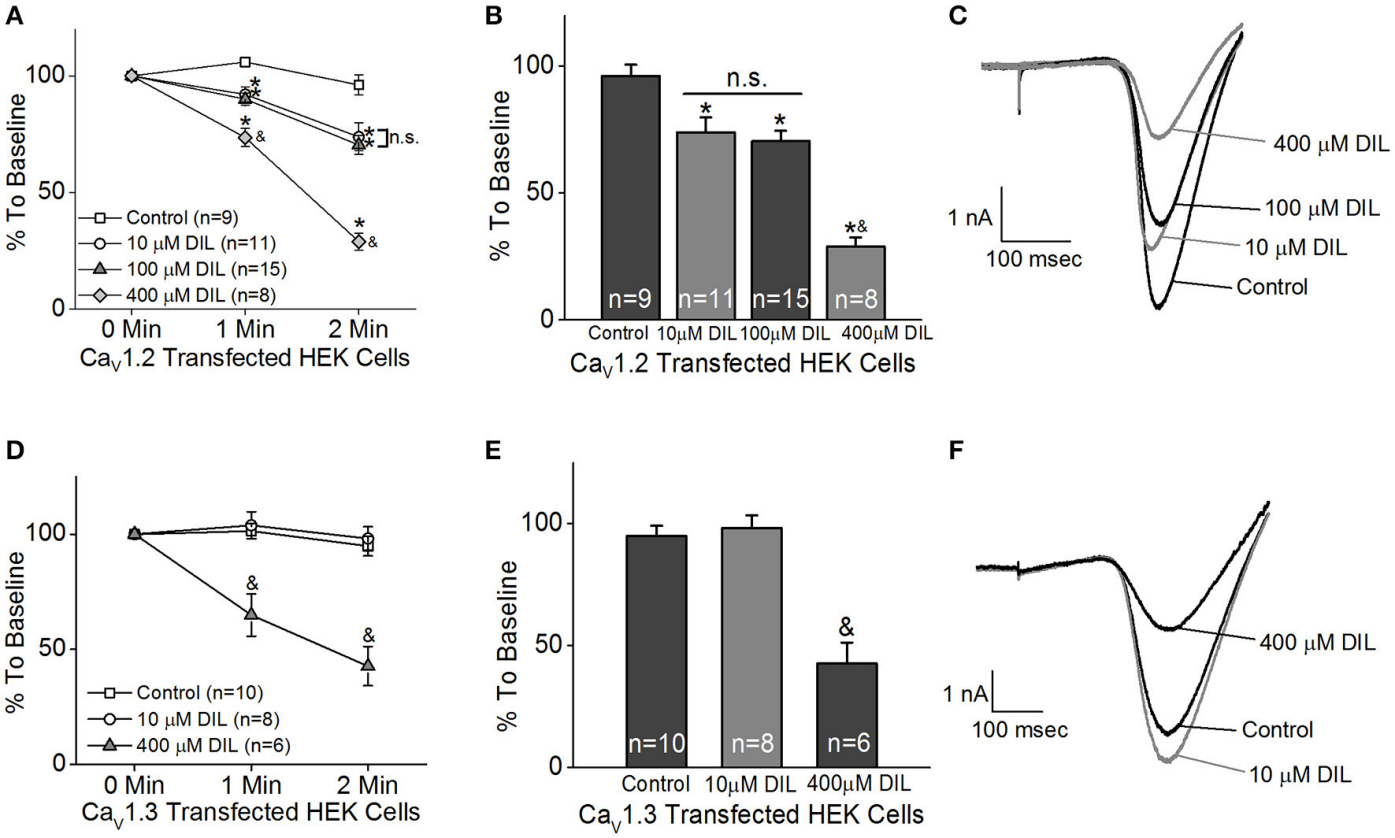
Different concentrations of diltiazem (DIL) are able to differentiate Ca_v_1.2-LTCC from Ca_v_1.3-LTCC currents in cultured HEK cells. The LTCC currents were recorded from Ca_v_1.2-transfected HEK cells **(A–C)** and Ca_v_1.3-transfected HEK cells **(D–F)** under the whole-cell patch-clamp configuration. **(A)** Perfusion with DIL at 10, 100, or 400 μM significantly decreases Ca_v_1.2-LTCC currents recorded from HEK cells transfected with Ca_v_1.2. **(B)** Perfusion with 10, 100, or 400 μM DIL for 2 min causes a reduction of Ca_v_1.2-LTCC currents. **(A,B)** The asterisk (^*^) indicates a statistically significant difference between the control and the 10, 100, and 400 μM DIL groups; “&” indicates that the 400 μM DIL group is statistically different from the other 3 groups; “n.s.” indicates that there is no statistical difference between the 10 and 100 μM DIL groups. **(C)** Representative traces recorded from the Ca_v_1.2-transfected HEK cells perfused with 0 (control), 10, 100, or 400 μM DIL are shown. **(D)** Diltiazem (DIL) at 400 μM, but not at 10 μM, causes a decrease in Ca_v_1.3-LTCC currents recorded from HEK cells transfected with Ca_v_1.3. **(E)** Perfusion with 400 μM DIL for 2 min causes a reduction of Ca_v_1.3-LTCC currents by more than 50%. **(D,E)** “&” indicates that the 400 μM DIL group is statistically significant from the control and the 10 μM DIL group. **(F)** Representative traces recorded from the Ca_v_1.3-transfected HEK cells perfused with 0 (control), 10, or 400 μM DIL are shown. ^*, &^*p* < 0.05.

The HEK 293 cells were co-transfected with Ca_v_1.2 or Ca_v_1.3 and β2+α2δ1 auxiliary subunits. Ba^2+^ currents were recorded in the absence or presence of DIL. After 5 min of perfusion, the control cells (extracellular solution added H_2_O, the vehicle) had a decrease of 20% from the baseline, which was due to the run-down of the current. After 5 min of perfusion with extracellular solution containing DIL (10, 100, or 400 μM), the current amplitudes all decreased to <50% of baseline (data not shown). Since perfusion for 2 min did not elicit any current run-down in the control cells, we chose to compare the inhibitory effects of DIL after 2 min of perfusion to avoid current run-down issues. Extracellular perfusion with 10 or 100 μM DIL for 2 min significantly decreased the Ca_v_1.2-LTCC currents by 27 and 30%, respectively, while perfusion of 400 μM DIL for 2 min further decreased Ca_v_1.2 currents by 71% (Figures 1A–C; Control: 96 ± 4%; DIL 10 μM: 73 ± 6%; DIL 100 μM: 70 ± 4%; DIL 400 μM: 29 ± 4%). However, extracellular perfusion with 10 μM DIL did not inhibit Ca_v_1.3-LTCC currents, while perfusion with 400 μM DIL did (Figures 1D–F; Control: 95 ± 4%; DIL 10 μM: 98 ± 5%; DIL 400 μM: 42 ± 9%). This result indicates that Ca_v_1.2 is more sensitive to DIL inhibition compared to Ca_v_1.3 in transfected HEK cells, so treatments with 10 μM DIL could selectively dampen Ca_v_1.2 with minimal impact on Ca_v_1.3.

### Inhibition of LTCCs decreases retinal light sensitivities

The ERG has long been used to determine overall retinal light sensitivities (Newman and Odette, 1984). The ERG a-wave reflects the photoreceptor responses to light stimulation, while the ERG b-wave represents the secondary light-evoked inner retinal responses, which reflects the summation from photoreceptor-bipolar cell synaptic transmission as well as responses from bipolar, amacrine, and Müller cells (Newman and Odette, 1984; Pinto et al., 2007). We employed the *ex vivo* transretinal ERG technique originally developed by Dr. Vladimir J. Kefalov (Vinberg et al., 2014), which allowed us to further dissect the contributions of LTCCs in retinal light responses from isolated mouse retinas. These *ex vivo* ERG recordings measure the light-induced voltage changes across the isolated retina from the photoreceptors to the ganglion cell layer (Vinberg et al., 2014), and it significantly improves the signal-to-noise ratio compared to *in vivo* ERGs and allows for easy assessments of pharmacological treatments in the isolated retina through extracellular perfusion (Kolesnikov and Kefalov, 2012; Vinberg et al., 2014).

**Figure 2:**
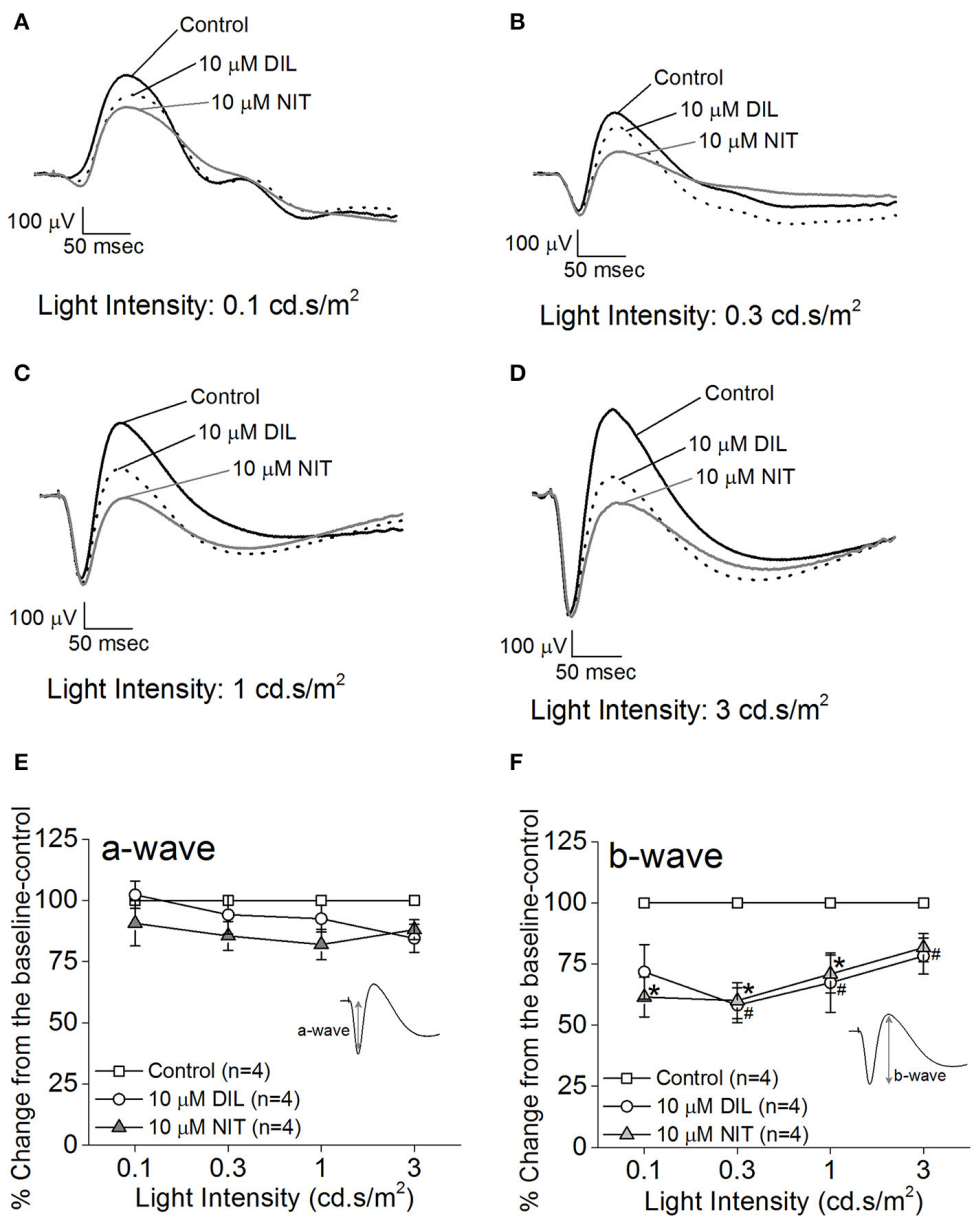
Differential contributions of LTCCs to retinal light responses as measured by *ex vivo* ERGs. Mice were dark adapted for at least 3 h, and the retinas were excised and placed in an *ex vivo* ERG recording chamber. The ERG responses were recorded under 4 different light intensities: 0.1, 0.3, 1, and 3 cd·s/m^2^. The ERG recordings were performed with normal perfusion buffer (Control), followed by perfusion with 10 μM DIL to inhibit Ca_v_1.2, and subsequently perfused with 10 μM nitrendipine (NIT) to block both Ca_v_1.2 and Ca_v_1.3. **(A–D)** Representative ERG waveforms recorded in different solutions (control, 10 μM DIL, and 10 μM NIT) are shown, which were recorded under light intensities of 0.1, 0.3, 1, and 3 cd·s/m^2^, respectively. **(E)** Perfusion with 10 μM DIL or 10 μM NIT for 10 min did not have significant effect on the ERG a-wave amplitudes. **(F)** Perfusion with 10 μM DIL or 10 μM NIT for 10 min decreased ERG b-wave amplitudes. The asterisk (^*^) indicates a statistically significant difference between the control and the 10 μM NIT group; “#” indicates that the 10 μM DIL group is statistically different from the control. ^*, #^*p* < 0.05.

Both retinas were isolated from a dark-adapted mouse and placed in a dual-recording chamber. The *ex vivo* ERG responses elicited at various light intensity flashes were recorded before and after perfusion with LTCC blockers under dim red lighting. We first tested the inhibitory effect of DIL and found that perfusion with 10 μM DIL for 10 min reached its maximal inhibition of the ERG responses, since continuous perfusion at this concentration for 30 min, or perfusion with higher concentrations of DIL (20, 50, or 100 μM), did not further decrease the ERG amplitudes. After ERG responses were first recorded under normal perfusion buffer, 10 μM DIL was perfused for 10 min to inhibit Ca_v_1.2 followed by another round of ERG recordings. Subsequently, after perfusion with 10 μM nitrendipine (NIT) for another 10 min to further inhibit both Ca_v_1.2 and Ca_v_1.3, a third round of ERG responses were recorded. The representative *ex vivo* ERG waveforms are presented in Figures 2A–D. We found that both DIL and NIT did not significantly affect the ERG a-wave responses (Figures 2E). Inhibition of LTCCs with DIL or NIT largely decreased the ERG b-wave responses (Figures 2F), indicating that Ca_v_1.2 and Ca_v_1.3 were involved in post-photoreceptor and inner retinal light responses.

**Figure 3:**
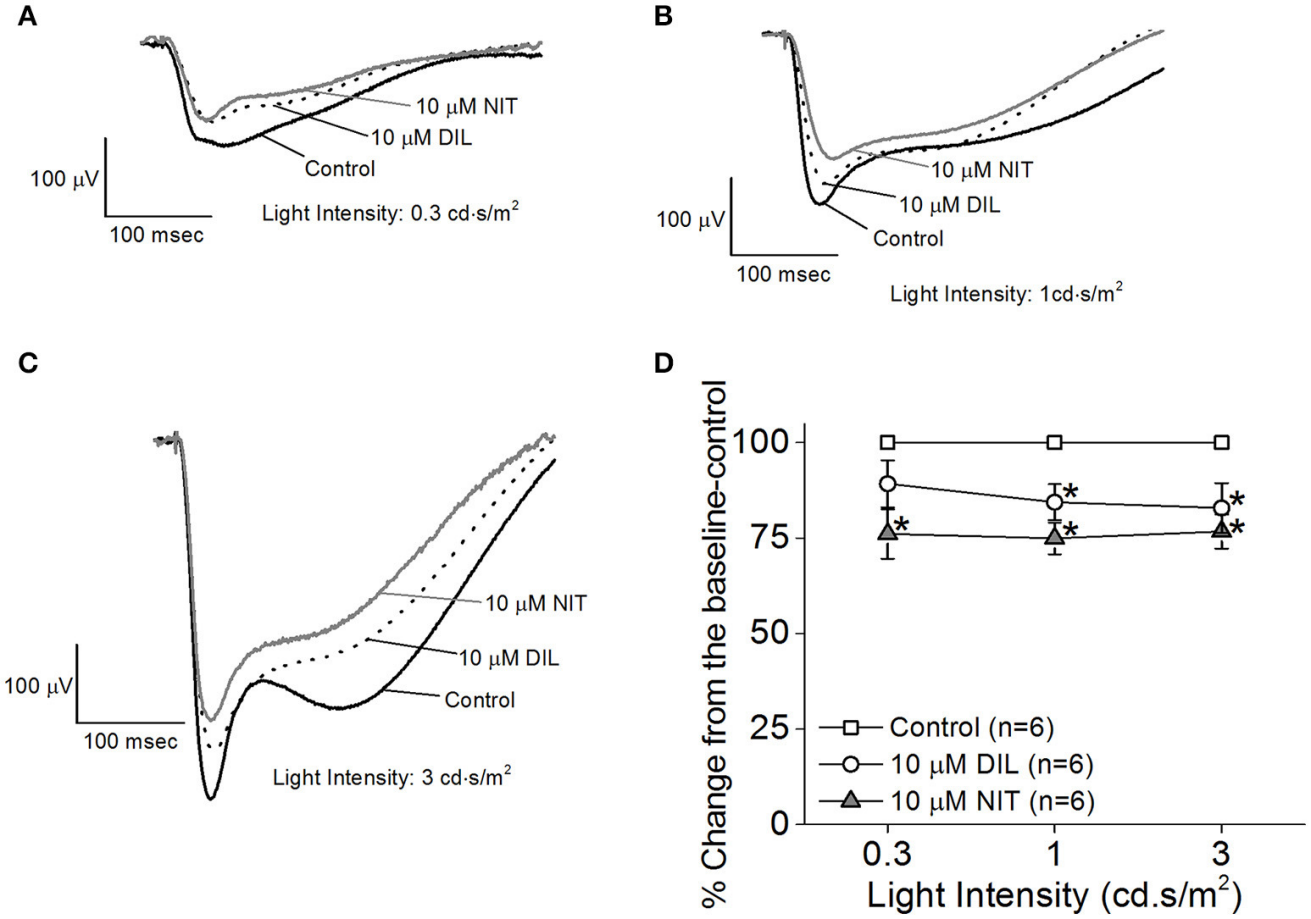
Differential contributions of LTCCs in *ex vivo* ERG a-wave responses. Mice were dark adapted for at least 3 h, and the retinas were excised and placed in an *ex vivo* ERG recording chamber. The ERG a-wave (photoreceptor) responses were isolated with a perfusion solution containing 2 mM L-glutamate and 10 μM DL-AP-4 and an electrode solution containing 2 mM L-glutamate and 10 mM BaCl_2_ to block the higher order of retinal photo-responses. The photoreceptor light responses were recorded under 3 different light intensities: 0.3, 1, and 3 cd·s/m^2^. The ERG recordings were first done with normal perfusion buffer (Control), followed by perfusion with 10 μM DIL, then perfusion with 10 μM NIT. **(A–C)** Representative ERG a-waves recorded in different solutions (control, 10 μM DIL, and 10 μM NIT) are shown, under light intensities of 0.3, 1, and 3 cd·s/m^2^, respectively. **(D)** Perfusion with 10 μM DIL and 10 μM NIT for 10 min each decreased ERG a-wave amplitudes. The asterisk (^*^) indicates that the 10 μM DIL or 10 μM NIT group is significantly different from the control. ^*^p < 0.05.

While the ERG a-wave represents the photoreceptor light responses, under higher light intensity stimulations, the a-wave is often contaminated by the rising phase of the b-wave responses. Without pharmacological isolation of the a-wave, there was no specific effect elicited by NIT or DIL determined. In order to assess the role of LTCCs in photoreceptor light responses, we isolated the photoreceptor responses (ERG a-wave) with a perfusion solution containing 2 mM L-glutamate and 10 μM DL-AP-4 and an electrode solution containing 2 mM L-glutamate and 10 mM BaCl_2_ to block the higher order retinal light responses (Kolesnikov and Kefalov, 2012). After a-wave isolation, we found that both DIL (10 μM) and NIT (10 μM) were able to reduce the ERG a-wave amplitudes but NIT caused a larger decrease (Figures 3A–D), indicating that both Ca_v_1.2 and Ca_v_1.3 were involved in photoreceptor light-evoked responses. In addition, at a lower light intensity stimulation (0.3 cd.s/m^2^), DIL did not significantly affect the ERG a-wave as it did at higher light intensity stimulations (1 and 3 cd.s/m^2^), but NIT significantly decreased the ERG a-wave at all three light intensity stimulations. This suggests that Ca_v_1.3 in photoreceptors might be more sensitive to changes in ambient light intensities, which echoes the notion that Ca_v_1.3 is “plastic” in responding to external stimulations. Since NIT and DIL were able to dampen isolated a-wave, this could partially explain the reduction of *ex vivo* ERG b-wave by NIT and DIL shown in Figure 2.

### Retinal light responses are decreased in both Ca_v_1.3^+/−^ and Ca_v_1.3^−/−^ mice

**Figure 4:**
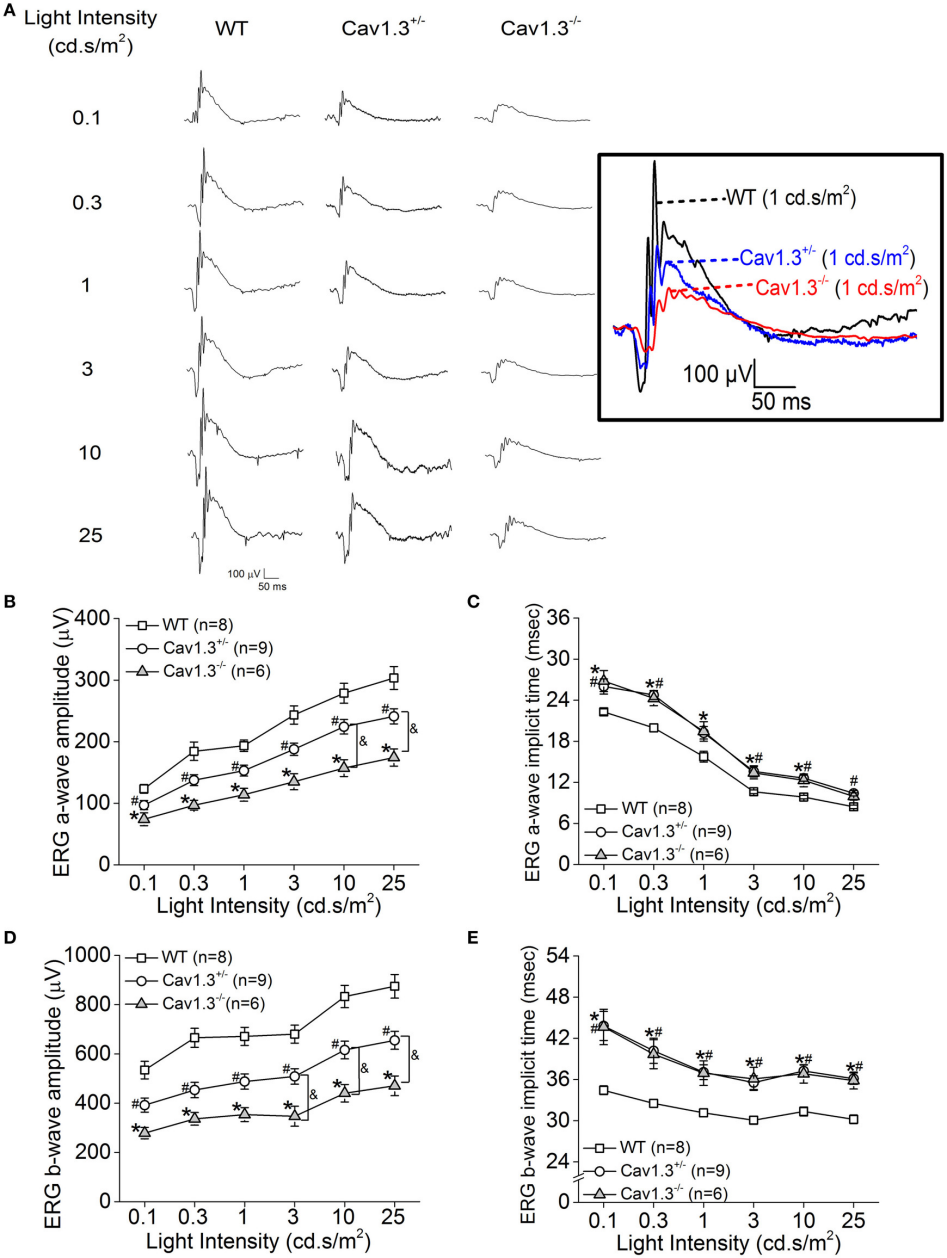
Retinal light responses are decreased in Ca_v_1.3^+/−^ and Ca_v_1.3^−/−^ mutant mice. The scotopic ERG amplitudes are decreased and implicit times delayed in both Ca_v_1.3^+/−^ (heterozygous mutant) and Ca_v_1.3^−/−^ (homozygous mutant) mice compared to the wild type (WT) mice. All mice were dark adapted for at least 8 h. Retinal light responses were measured when mice were exposed to a series of light intensities from 0.1 to 25 cd·s/m^2^. **(A)** Representative ERG wave forms recorded from WT (left), Ca_v_1.3^+/−^ (middle), and Ca_v_1.3^−/−^ (right) mouse eyes in response to each stimulating light intensity. The superimposed ERG traces from the WT (black), Ca_v_1.3^+/−^ (blue), and Ca_v_1.3^−/−^ (red) mice elicited at 1 cd.s/m^2^ light intensity are shown in the box. **(B)** The average ERG a-wave amplitudes are decreased in Ca_v_1.3^+/−^ and Ca_v_1.3^−/−^ mice compared to the WT. **(C)** The average ERG a-wave implicit times are increased in Ca_v_1.3^+/−^ and Ca_v_1.3^−/−^ mice compared to the WT. **(D)** The average ERG b-wave amplitudes are decreased in Ca_v_1.3^+/−^ and Ca_v_1.3^−/−^ mice compared to the WT. **(E)** The average ERG b-wave implicit times are increased in Ca_v_1.3^+/−^ and Ca_v_1.3^−/−^ mice compared to the WT. The asterisk (^*^) indicates a statistically significant difference between the WT and Ca_v_1.3^−/−^ mice; “#” indicates a statistically significant difference between the WT and Ca_v_1.3^+/−^ mice; “&” indicates a statistically significant difference between the Ca_v_1.3^+/−^ and Ca_v_1.3^−/−^ mice. ^*, #, &^*p* < 0.05.

Since all Ca_v_ (Ca_v_1.2, 1.3, and 1.4) channels are expressed in the retina, our pharmacological studies (Figures 2, 3) could not exclude the possibility that DIL and NIT might inhibit Ca_v_1.4. Thus, to further verify the role of Ca_v_1.3 in retinal light sensitivities, we recorded retinal light responses using *in vivo* ERG recordings from Ca_v_1.3^−/−^ homozygous null, Ca_v_1.3^+/−^ heterozygous, and wild type (WT) mice at 2.5 months old. Mice were dark adapted overnight for at least 8 h prior to ERG recordings with various light intensities at 0.1, 0.3, 1, 3, 10, and 25 cd·s/m^2^ (Figure 4A). There were 3 Ca_v_1.3^+/+^ (WT) mice from the littermates and 5 WT mice purchased from the vendor. There was no statistical difference in the ERG amplitudes and implicit times recorded between these two WT groups, so we merged the data as a single WT group. But both Ca_v_1.3^+/−^ and Ca_v_1.3^−/−^ mice had significantly decreased ERG a-wave amplitudes (Figure 4B) and delayed implicit times (Figure 4C) compared to that of WT mice. Similarly, the ERG b-wave amplitudes were decreased (Figure 4D) and implicit times delayed (Figure 4E) in Ca_v_1.3^+/−^ and Ca_v_1.3^−/−^ mice compared to WT mice (Table 1).

**Table 1:** Dark-adapted retinal light responses (Data for Figures 4B–E).

**Light intensity (cd·s/m^2^)**	**a-wave amplitude (μV)**			**a-wave implicit time (ms)**		
	**WT**	**Ca_v_1.3^+/−^**	**Ca_v_1.3^−/−^**	**WT**	**Ca_v_1.3^+/−^**	**Ca_v_1.3^−/−^**
0.1	123.4 ± 8.0	96.9 ± 7.5	74.0 ± 10.4^*^ ^*^p = 0.001	22.3 ± 0.4	26.0 ± 1.1^#^ ^#^*p* = 0.037	26.8 ± 1.5^*^ ^*^p = 0.020
0.3	184.4 ± 15.0	137.5 ± 8.8^#^ ^#^*p* = 0.01	96.8 ± 8.1^*^^&^ ^*^p = 0.00002 ^&^*P* = 0.042	20.0 ± 0.6	24.8 ± 0.7^#^ ^#^*p* = 0.00008	24.3 ± 1.1^*^ ^*^p = 0.001
1	193.4 ± 9.2	153.0 ± 8.8^#^ ^#^*p* = 0.008	113.9 ± 10.4^*^ ^*^p = 0.000004, ^&^*P* = 0.017	15.8 ± 0.8	19.2 ± 0.9^#^ ^#^*P* = 0.040	19.4 ± 1.4
3	243.4 ± 14.8	187.7 ± 9.9^#^ ^#^*p* = 0.006	134.9 ± 13.1^*^^&^ ^*^p = 0.000002, ^&^*P* = 0.015	10.6 ± 0.3	13.6 ± 0.8^#^ ^#^*p* = 0.011	13.4 ± 0.9^*^ ^*^p = 0.036
10	278.7 ± 16.2	224.3 ± 11.7^#^ ^#^*p* = 0.016	157.2 ± 13.7^*^^&^ ^*^p = 0.000002, ^&^*p* = 0.005	9.8 ± 0.3	12.6 ± 0.5^#^ ^#^*p* = 0.003	12.3 ± 0.9^*^ ^*^p = 0.015
25	303.5 ± 18.6	241.2 ± 12.3^#^ ^#^*p* = 0.011	174.4 ± 14.0^*^^&^ ^*^p = 0.000003, ^&^*p* = 0.01	8.4 ± 0.3	10.4 ± 0.4^#^ ^#^*p* = 0.002	9.9 ± 0.5^*^ ^*^p = 0.036
**Light intensity (cd·s/m^2^)**	**b-wave amplitude (μV)**			**b-wave implicit time (ms)**		
	**WT**	**Ca_v_1.3^+/−^**	**Ca_v_1.3^−/−^**	**WT**	**Ca_v_1.3^+/−^**	**Ca_v_1.3^−/−^**
0.1	534.5 ± 35.6	392.2 ± 28.7^#^ ^#^*p* = 0.004	278.6 ± 23.2^*^^&^ ^*^p = 0.000005, ^&^*p* = 0.035	34.4 ± 0.6	43.8 ± 2.2^#^ ^#^*p* = 0.003	43.7 ± 2.6^*^ ^*^p = 0.007
0.3	665.6 ± 38.6	453.6 ± 31.2^#^ ^#^*p* = 0.00008	336.5 ± 25.8^*^^&^ ^*^p = 0.0000001 ^&^*p* = 0.049	32.5 ± 0.6	40.2 ± 1.8^#^ ^#^*p* = 0.004	39.7 ± 2.1^*^ ^*^p = 0.015
1	671.3 ± 36.9	488.0 ± 30.9^#^ ^#^*p* = 0.0005	353.7 ± 28.4^*^ ^*^p = 0.0000002, ^&^*p* = 0.019	31.1 ± 0.6	37.1 ± 1.1^#^ ^#^*p* = 0.001	36.9 ± 1.8^*^ ^*^p = 0.005
3	680.4 ± 36.5	508.8 ± 31.4^#^ ^#^*p* = 0.003	347.1 ± 40.7^*, &^ ^*^p = 0.0000005, ^&^*p* = 0.008	30.1 ± 0.5	35.5 ± 1.0^#^ ^#^*p* = 0.001	36.1 ± 1.7^*^ ^*^p = 0.002
10	833.0 ± 45.4	616.1 ± 35.9^#^ ^#^*p* = 0.0007	440.5 ± 35.5^*, &^ ^*^p = 0.0000001, ^&^*p* = 0.010	31.3 ± 0.7	37.3 ± 0.9^#^ ^#^*p* = 0.0001	36.8 ± 1.3^*^ ^*^p = 0.001
25	874.7 ± 47.9	655.3 ± 36.4^#^ ^#^*p* = 0.001	470.8 ± 39.9^*, &^ ^*^p = 0.0000002, ^&^*p* = 0.010	30.2 ± 0.6	36.1 ± 0.8^#^ ^#^*p* = 0.00004	35.9 ± 1.2^*^ ^*^p = 0.0004

* *Denotes Ca_v_1.3^−/−^ significantly different from WT*.

# *Denotes Ca_v_1.3^+/−^ significantly different from WT*.

& *Denotes Ca_v_1.3^−/−^ significantly different from Ca_v_1.3^+/−^*.

Significance was determined when one-way ANOVA Tukey post hoc tests achieved ^*^p < 0.05.

**Figure 5:**
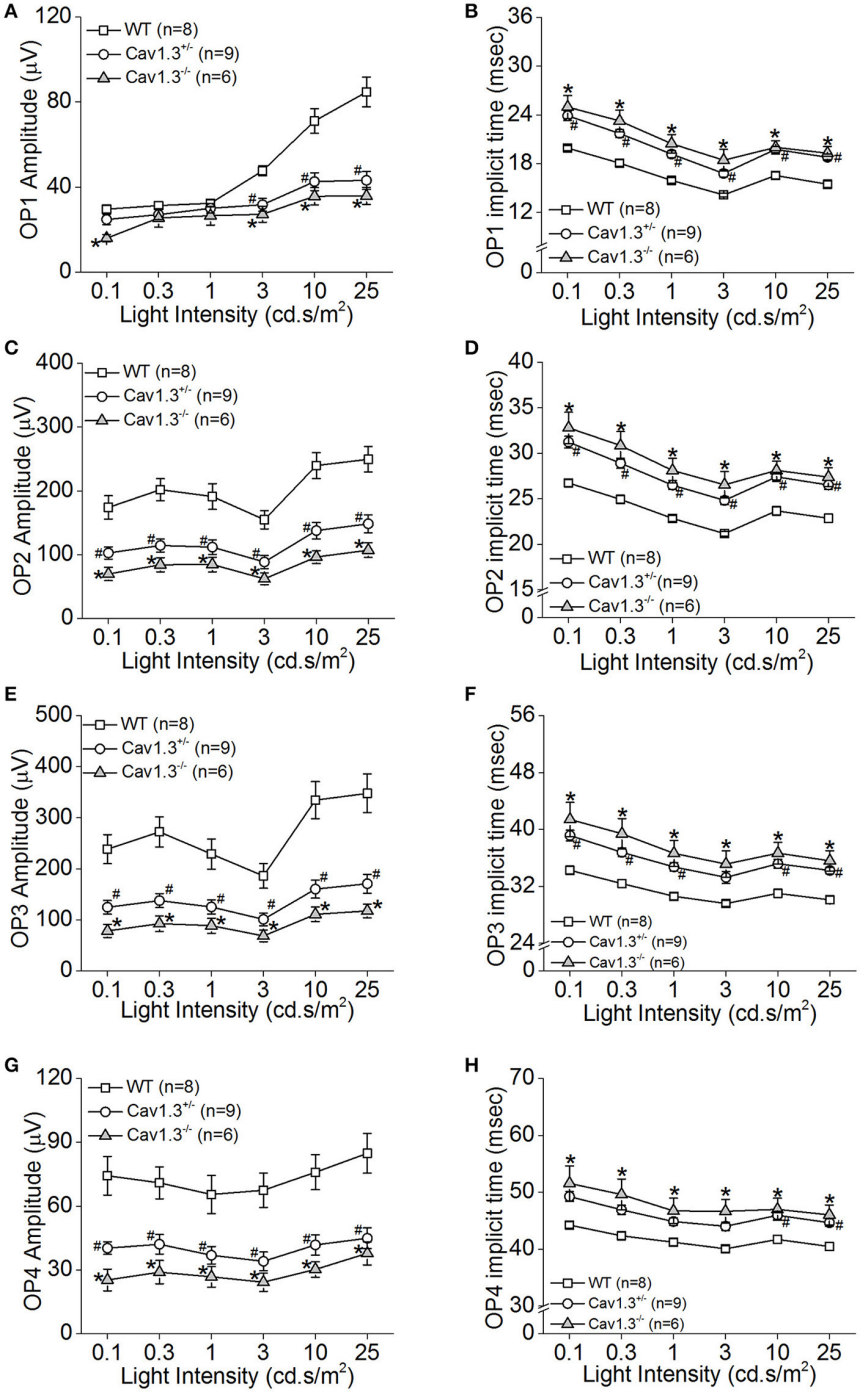
The inner retinal light responses are decreased in Ca_v_1.3^+/−^ and Ca_v_1.3^−/−^ mutant mice. The oscillatory potential (OP) amplitudes are decreased and implicit times delayed in both Ca_v_1.3^+/−^ and Ca_v_1.3^−/−^ mice compared to the wild type (WT). The OPs were analyzed after the scotopic ERG responses were filtered between 100 and 300 Hz band-pass using the ERGView4.4 software (Ocuscience). **(A,B)** The average OP1 amplitudes **(A)** are decreased, but the implicit time **(B)** is increased in Ca_v_1.3^+/−^ and Ca_v_1.3^−/−^ mice compared to the WT. **(C,D)** The average OP2 amplitudes **(C)** are decreased, but the implicit time **(D)** is increased in Ca_v_1.3^+/−^ and Ca_v_1.3^−/−^ mice compared to the WT. **(E,F)** The average OP3 amplitudes **(E)** are decreased, but the implicit time **(F)** is increased in Ca_v_1.3^+/−^ and Ca_v_1.3^−/−^ mice compared to the WT. **(G,H)** The average OP4 amplitudes **(G)** are decreased, but the implicit time **(H)** is increased in Ca_v_1.3^+/−^ and Ca_v_1.3^−/−^ mice compared to the WT. The asterisk (^*^) indicates a statistically significant difference between the WT and Ca_v_1.3^−/−^ mice; “#” indicates a statistically significant difference between the WT and Ca_v_1.3^+/−^ mice. ^*, #^*p* < 0.05.

We further analyzed the ERG oscillatory potentials (OP1-OP4), which largely represent the inner retinal responses especially from amacrine cells (Wachtmeister and Dowling, 1978; Wachtmeister, 1998; Pinto et al., 2007). Both Ca_v_1.3^+/−^ and Ca_v_1.3^−/−^ mice had decreased OP1-4 amplitudes (Figures 5A,C,E,G) and delayed OP1-4 implicit times (Figures 5B,D,F,H) compared to WT mice (Table 2). Hence, these *in vivo* ERG data clearly demonstrate that Ca_v_1.3 contributes to both outer and inner retinal light responses.

**Table 2:** Oscillatory Potential Responses (Data for Figures 5A–H).

**Light intensity (cd·s/m^2^)**	**OP1 amplitude (μV)**			**OP1 implicit time (ms)**		
	**WT**	**Ca_v_1.3^+/−^**	**Ca_v_1.3^−/−^**	**WT**	**Ca_v_1.3^+/−^**	**Ca_v_1.3^−/−^**
0.1	29.7 ± 1.7	24.8 ± 2.4	16.0 ± 1.7^*^ ^*^p = 0.0002	19.9 ± 0.3	23.9 ± 0.6^#^ ^#^*p* = 0.004	25.0 ± 1.4^*^ ^*^p = 0.0005
0.3	31.3 ± 1.9	27.1 ± 2.4	25.5 ± 4.3	18.0 ± 0.4	21.7 ± 0.5^#^ ^#^*p* = 0.003	23.3 ± 1.3^*^ ^*^p = 0.0001
1	32.4 ± 1.6	30.0 ± 2.3	26.6 ± 4.4	15.9 ± 0.4	19.2 ± 0.4^#^ ^#^*p* = 0.003	20.5 ± 1.1^*^ ^*^p = 0.0001
3	47.7 ± 2.5	31.7 ± 3.0^#^ ^#^*p* = 0.002	27.3 ± 3.7^*^ ^*^p = 0.0002	14.2 ± 0.3	16.8 ± 0.4^#^ ^#^*p* = 0.041	18.4 ± 1.3^*^ ^*^p = 0.001,
10	71.1 ± 5.8	42.6 ± 4.2^#^ ^#^*p* = 0.0003	35.7 ± 4.1^*^ ^*^p = 0.00003	16.6 ± 0.4	19.8 ± 0.4^#^ ^#^*p* = 0.0003	20.0 ± 0.8^*^ ^*^p = 0.0003
25	84.7 ± 7.0	43.2 ± 4.1^#^ ^#^*p* = 0.000003	35.9 ± 3.9^*^ ^*^p = 0.0000005	15.5 ± 0.4	18.8 ± 0.4^#^ ^#^*p* = 0.0001	19.3 ± 0.8^*^ ^*^p = 0.00005
**Light intensity (cd·s/m^2^)**	**OP2 amplitude (μV)**			**OP2 implicit Time (ms)**		
	**WT**	**Ca_v_1.3^+/−^**	**Ca_v_1.3^−/−^**	**WT**	**Ca_v_1.3^+/−^**	**Ca_v_1.3^−/−^**
0.1	174.1 ± 18.3	102.7 ± 9.5^#^ ^#^*p* = 0.001	69.9 ± 10.5^*^ ^*^p = 0.00001	26.7 ± 0.4	31.2 ± 0.6^#^ ^#^*p* = 0.005	32.8 ± 1.7^*^ ^*^p = 0.0004
0.3	201.9 ± 17.2	114.4 ± 10.3^#^ ^#^*p* = 0.00006	84.4 ± 11.1^*^ ^*^p = 0.000001	24.9 ± 0.4	28.9 ± 0.5^#^ ^#^*p* = 0.005	30.9 ± 1.5^*^ ^*^p = 0.0001
1	191.2 ± 20.3	111.9 ± 11.2^#^ ^#^*p* = 0.001	84.7 ± 11.5^*^ ^*^p = 0.000006	22.9 ± 0.3	26.5 ± 0.5^#^ ^#^*p* = 0.003	28.1 ± 1.3^*^ ^*^p = 0.00009
3	154.8 ± 14.8	88.7 ± 10.3^#^ ^#^*p* = 0.0006	62.3 ± 9.0^*^ ^*^p = 0.00001	21.1 ± 0.3	24.8 ± 0.4^#^ ^#^*p* = 0.006	26.5 ± 1.5^*^ ^*^p = 0.0002
10	239.8 ± 20.2	137.8 ± 13.1^#^ ^#^*p* = 0.00006	96.5 ± 10.1^*^ ^*^p = 0.00005	23.7 ± 0.5	27.4 ± 0.5^#^ ^#^*p* = 0.0006	28.1 ± 1.0^*^ ^*^p = 0.0002
25	249.5 ± 20.1	148.5 ± 14.1^#^ ^#^*p* = 0.0001	107.1 ± 11.4^*^ ^*^p = 0.000001	22.9 ± 0.5	26.5 ± 0.5^#^ ^#^*p* = 0.0008	27.4 ± 1.0^*^ ^*^p = 0.0001
**Light intensity (cd·s/m^2^)**	**OP3 amplitude (μV)**			**OP3 implicit Time (ms)**		
	**WT**	**Ca_v_1.3^+/−^**	**Ca_v_1.3^−/−^**	**WT**	**Ca_v_1.3^+/−^**	**Ca_v_1.3^−/−^**
0.1	238.6 ± 28.5	124.8 ± 13.9^#^ ^#^*p* = 0.0006	78.4 ± 12.7^*^ ^*^p = 0.000008	34.3 ± 0.5	39.2 ± 0.7^#^ ^#^*p* = 0.025	41.4 ± 2.4^*^ ^*^p = 0.002
0.3	272.4 ± 29.5	137.9 ± 13.8^#^ ^#^*p* = 0.00008	92.6 ± 15.2^*^ ^*^p = 0.000002	32.4 ± 0.5	36.8 ± 0.6^#^ ^#^*p* = 0.024	39.4 ± 2.1^*^ ^*^p = 0.0006
1	229.0 ± 29.7	125.4 ± 14.1^#^ ^#^*p* = 0.002	88.8 ± 14.8^*^ ^*^p = 0.0001	30.6 ± 0.5	34.7 ± 0.6^#^ ^#^*p* = 0.014	36.6 ± 1.8^*^ ^*^p = 0.0007
3	186.2 ± 25.3	101.2 ± 12.50^#^ ^#^*p* = 0.003	68.7 ± 11.2^*^ ^*^p = 0.0001	29.6 ± 0.5	33.3 ± 0.8	35.1 ± 1.9^*^ ^*^p = 0.005
10	334.6 ± 36.2	160.4 ± 17.9^#^ ^#^*p* = 0.00003	111.0 ± 14.2^*^ ^*^p = 0.000001	31.0 ± 0.6	35.2 ± 0.6^#^ ^#^*p* = 0.006	36.7 ± 1.5^*^ ^*^p = 0.0004
25	347.8 ± 37.8	170.8 ± 18.5^#^ ^#^*p* = 0.0004	117.7 ± 13.2^*^ ^*^p = 0.000001	30.1 ± 0.6	34.2 ± 0.5^#^ ^#^*p* = 0.002	35.6 ± 1.4^*^ ^*^p = 0.0002
**Light intensity (cd·s/m^2^)**	**OP4 amplitude (μV)**			**OP4 implicit Time (ms)**		
	**WT**	**Ca_v_1.3^+/−^**	**Ca_v_1.3^−/−^**	**WT**	**Ca_v_1.3^+/−^**	**Ca_v_1.3^−/−^**
0.1	74.3 ± 9.1	40.3 ± 3.0^#^ ^#^*p* = 0.0007	25.3 ± 5.1^*^ ^*^p = 0.00001	44.2 ± 0.6	49.3 ± 0.8	51.6 ± 3.1^*^ ^*^p = 0.013
0.3	70.9 ± 7.5	42.1 ± 4.6^#^ ^#^*p* = 0.003	29.0 ± 5.4^*^ ^*^p = 0.00007	42.3 ± 0.6	46.9 ± 0.8	49.6 ± 2.7^*^ ^*^p = 0.006
1	65.5 ± 9.0	36.9 ± 4.1^#^ ^#^*p* = 0.006	26.8 ± 4.8^*^ ^*^p = 0.0005	41.2 ± 0.6	44.8 ± 0.6	46.7 ± 2.2^*^ ^*^p = 0.01
3	67.5 ± 8.3	34.1 ± 4.4^#^ ^#^*p* = 0.0005	24.3 ± 4.3^*^ ^*^p = 0.00004	40.1 ± 0.6	44.0 ± 0.7	46.7 ± 2.1^*^ ^*^p = 0.002
10	75.9 ± 8.2	41.8 ± 4.7^#^ ^#^*p* = 0.0005	30.3 ± 3.6^*^ ^*^p = 0.00002	41.7 ± 0.7	45.9 ± 0.7^#^ ^#^*p* = 0.025	47.0 ± 1.9^*^ ^*^p = 0.008
25	84.8 ± 9.4	44.9 ± 4.9^#^ ^#^*p* = 0.0004	37.8 ± 5.4^*^ ^*^p = 0.0001	40.5 ± 0.7	44.7 ± 0.7^#^ ^#^*p* = 0.015	46.0 ± 1.8^*^ ^*^p = 0.002

* *Denotes Ca_v_1.3^−/−^ significantly different from WT*.

# *Denotes Ca_v_1.3^+/−^ significantly different from WT*.

Significance was determined when one-way ANOVA Tukey post hoc tests achieved ^*^p < 0.05.

### Deletion of Ca_v_1.3 α1 subunit affects the density of ribbon synapses in the retinal outer plexiform layer (OPL)

**Figure 6:**
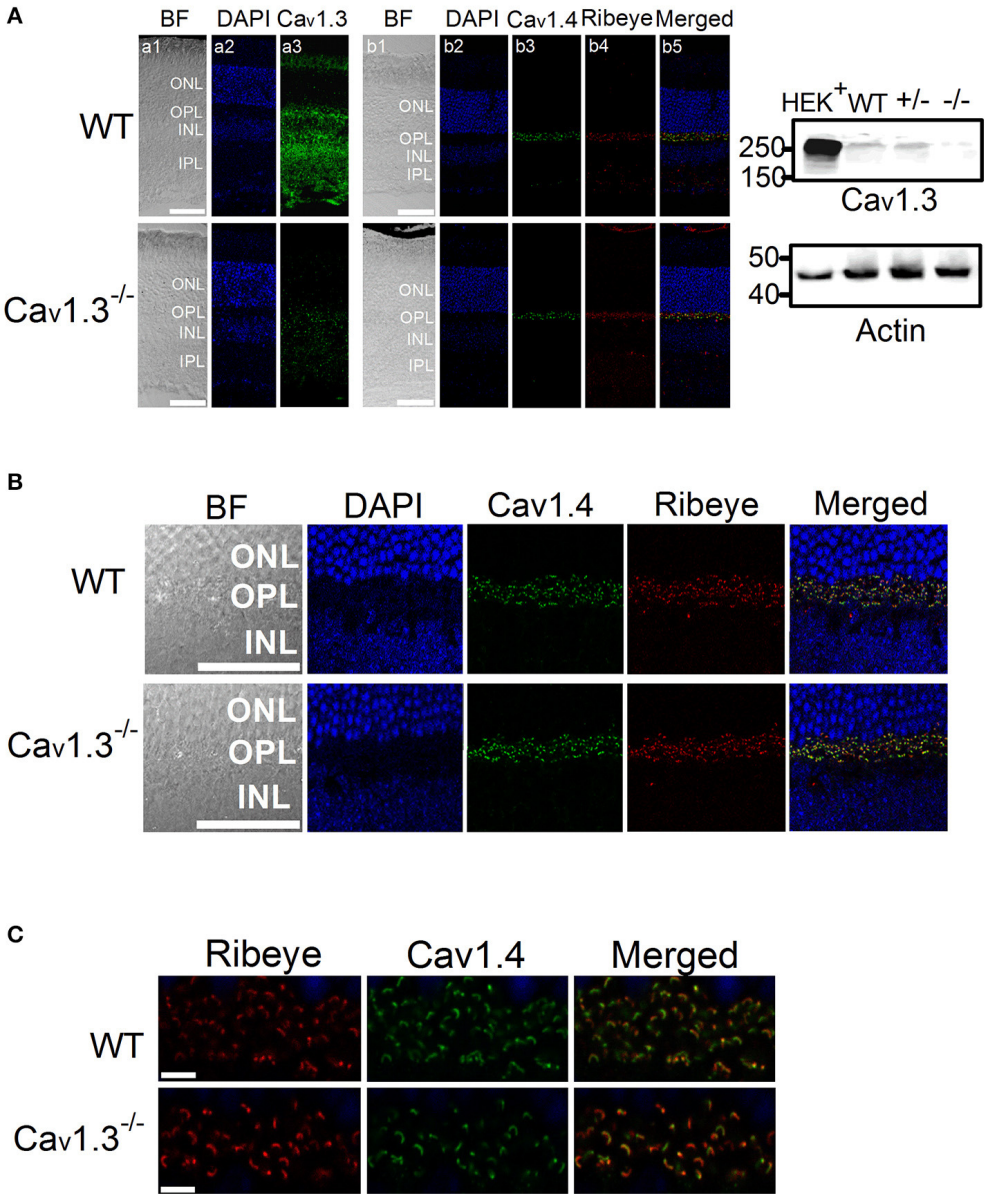
Immunofluorescent changes of Ca_v_1.3, Ca_v_1.4, and Ribeye in Ca_v_1.3^−/−^ mouse retinas. Ca_v_1.3^+/+^ (WT) and Ca_v_1.3^−/−^ retinal sections (10 μm) were stained for Ca_v_1.3, Ca_v_1.4, and Ribeye. **(A)** Representative images at a lower magnification (20 X) of WT (upper panel) and Ca_v_1.3^−/−^ (lower panel) retinal sections. DAPI stains the cell nucleus. BF: bright field; ONL: outer nuclear layer; OPL: outer plexiform layer; INL: inner nuclear layer; IPL: inner plexiform layer. The scale bar = 50 μm. Right panel: The Western blots from Ca_v_1.3 transfected HEK cells (HEK+), Ca_v_1.3^+/+^ (WT) retina, Ca_v_1.3^+/−^ retina, and Ca_v_1.3^−/−^ retina show the protein band of Ca_v_1.3 at ~250 kD. Actin serves as the loading controls. **(B)** Representative images at a higher magnification (40 X) of WT and Ca_v_1.3^−/−^ retinal sections stained for Ca_v_1.4 and Ribeye. The scale bar = 50 μm. **(C)** Fluorescent images focused on the OPL at a higher magnification (80 X) from WT and Ca_v_1.3^−/−^ retinal sections are shown. The scale bar = 5 μm. The images taken at this magnification were used for statistical analyses (Table 3).

Our ERG results revealed that deletion of Ca_v_1.3 had an impact on the retinal light responses, mainly on the b-wave amplitude, suggesting that the light signal from the photoreceptors to the inner retina, as well as the light responses in the inner retinal neurons were dampened. The Ca_v_1.3^−/−^ retina (−/−) had the least protein expression of Ca_v_1.3 compared to the WT or Ca_v_1.3^+/−^ (+/−) retina as shown by Western blots (Figure 6A, right panel, arrow head). Hence, we carried out immunostaining to examine any potential morphological changes in the Ca_v_1.3^−/−^ retina.

There was no noticeable morphological change of the overall retinal organization in the Ca_v_1.3^−/−^ retina compared to the WT (Figure 6A). In the WT retina, Ca_v_1.3 was present in the photoreceptor inner segment (IS), outer nuclear layer (ONL), OPL, inner nuclear layer (INL), inner plexiform layer (IPL), and the retinal ganglion cells (Figure 6A). Even though Ca_v_1.3 existed in the OPL, it was not exclusively co-localized with Ribeye at the ribbon synapses (Figure 6B), indicating that Ca_v_1.3 is present at the synaptic terminals but maybe not specifically at the ribbon structure. The Ca_v_1.3 fluorescence in the Ca_v_1.3^−/−^ retinal section could be the background staining.

Since one of the essential roles of LTCCs is governing the tonic release of neurotransmitters from photoreceptors, we focused on the OPL and detected two major proteins (Ribeye and Ca_v_1.4) that are present in the photoreceptor ribbon synapses. Ca_v_1.4 is largely expressed in the OPL, which contains the synaptic terminals of rod and cone photoreceptors (Figure 6A). Staining with Ribeye, ribbon synapses have a horseshoe-like shape in the OPL, and Ca_v_1.4 is mostly co-localized within the synaptic ribbons (Figure 6C), which is consistent with a previous report (Lee et al., 2015). We quantified both Ribeye positive and Ca_v_1.4 positive staining at the OPL using “Fiji” with “Coloc 2” to analyze their colocalization. “Fiji” is an image processing package that is an open-source platform for biological image analysis (Schindelin et al., 2012). The Li's Intensity Correlation Quotient (ICQ) value (Li et al., 2004) was generated in this software package to determine the degree of Ribeye and Ca_v_1.4 colocalization: for colocalized/dependent staining 0 < ICQ ≤ +0.5; ICQ = ~0 for random staining; for segregated staining 0 > ICQ ≥ −0.5. In WT and Ca_v_1.3^−/−^ mouse retinas, Ribeye and Ca_v_1.4 were highly colocalized, with Li's ICQ at ~0.35 for both. With further quantification using Image J to analyze the Ribeye or Ca_v_1.4 positive structure, we found that WT retinas had significantly higher Ribeye positive and Ca_v_1.4 positive structures at the OPL compared to that in the Ca_v_1.3^−/−^ mouse retinas (Table 3, Supplementary Figure 1). Thus, deletion of Ca_v_1.3 had a negative impact on the photoreceptor ribbon synapses.

**Table 3:** Analyses of synaptic structures at the OPL.

**Fiji Coloc2 analysis**	**WT (*n* = 5)**	**Ca_v_1.3^−/−^ (*n* = 5)**	***t*-test**
Li's ICQ for colocalization of Ribeye and Ca_v_1.4	0.352 ± 0.012	0.357 ± 0.007	*P* = 0.70
**Image J analysis of fluorescent positive structures at the OPL**			
Ribeye positive (pixels)	5,579.8 ± 522.60	4,011.6 ± 278.98	^*^p = 0.029
Ca_v_1.4 positive (pixels)	5,272.6 ± 660.42	3,164.2 ± 214.84	^*^p = 0.016

Significance was determined when the Student's t-test achieved

* *p < 0.05*.

## Discussion

In rod and cone photoreceptors, Ca_v_1.3 exists in the inner segments, cell bodies, and synaptic terminals (Firth et al., 2001; Xu et al., 2002; Morgans et al., 2005; Hull et al., 2006b; Cristofanilli et al., 2007; Ko et al., 2007). Even though there is no report on obvious visual deficiencies in animals or humans with Ca_v_1.3 mutations, in a previous study (Busquet et al., 2010), a failure to regulate Ca_v_1.3 is found in a mouse model of Usher syndrome, the most common cause of combined deafness and blindness in humans (Petit, 2001; Joiner and Lee, 2015). One Usher protein, USH2D (whirlin), is known to interact with Ca_v_1.3 in the retinal photoreceptors (Kersten et al., 2010). Hence, Ca_v_1.3 may play a role in retinal light responses.

We first used pharmacological blockers to identify the role of Ca_v_1.3 in retinal physiology and function. In previously published reports, the effectiveness of DIL on Ca_v_1.2 vs. Ca_v_1.3 was not compared in the same cell type or preparation (Cai et al., 1997; Hockerman et al., 2000; Schnee and Ricci, 2003; Baumann et al., 2004; Tarabova et al., 2007; Bissig et al., 2013; Berkowitz et al., 2014), so we set forth using HEK cells transfected with Ca_v_1.2 or Ca_v_1.3 and identified that DIL at 10 μM effectively inhibited Ca_v_1.2 but not Ca_v_1.3 (Figure 1). By applying 10 μM DIL or NIT in our *ex vivo* ERG studies, we found that both Ca_v_1.2 and Ca_v_1.3 contributed to retinal light responses (Figures 2, 3). Unfortunately, we could not completely distinguish the contribution of Ca_v_1.2 from Ca_v_1.3 in *ex vivo* retinal light responses, even though *ex vivo* ERG recordings have enhanced signal-to-noise ratios compared to *in vivo* ERGs and allow easy assessments of pharmacological treatments in the isolated retina (Kolesnikov and Kefalov, 2012; Vinberg et al., 2014). One possible explanation is that DIL and NIT might also affect Ca_v_1.4 in the retina. Thus, we next used a genetic strategy to verify the role of Ca_v_1.3 in retinal function.

We used Ca_v_1.3-null mice (Ca_v_1.3^−/−^) to further verify the role of Ca_v_1.3 in retinal light responses recorded by ERG. While the ERG a-wave reflects the photoreceptor light responses, the ERG b-wave represents the inner retinal light responses (Pinto et al., 2007), including the light-evoked depolarization of ON bipolar cells (Stockton and Slaughter, 1989) and amacrine cells, especially with the OP components of the b-wave reflecting the amacrine cell responses (Korol et al., 1975; Palmowski-Wolfe et al., 2006). Compared to the WT, the Ca_v_1.3^−/−^ mice had significantly dampened retinal light responses in both ERG a- and b-waves, as well as the OPs (Figures 4, 5 and Tables 1, 2). Interestingly, Ca_v_1.3 is expressed in the lobular appendages of AII amacrine cells (Habermann et al., 2003), and Ca_v_1.3 is responsible for glycine release from the AII amacrine cells (Balakrishnan et al., 2015). Thus, our ERG recordings from Ca_v_1.3 ^−/−^ mice with decreased OPs might reflect impaired crossover inhibition from amacrine cells (Menger et al., 1998; Habermann et al., 2003; Balakrishnan et al., 2015).

An alternative explanation is that the decreased OPs and b-wave in Ca_v_1.3^−/−^ mice were caused by impaired neurotransmission from photoreceptors to bipolar cells. Since our immunostaining showed that in Ca_v_1.3^−/−^ mouse retina, there was a significant decrease of Ca_v_1.4 positive ribbon synapses at the OPL. We previously showed that deletion of Ca_v_1.3 decreases the distribution of retinoschisin (RS1) in the retinal OPL (Shi et al., 2017). Retinoschisin is an extracellular adhesion protein mainly secreted from photoreceptors and bipolar cells (Reid et al., 1999, 2003; Reid and Farber, 2005). Mutations in the gene encoding RS1 cause X-linked juvenile retinoschisis that features disorganization of retinal cell layers, disruption of synaptic structures and neurotransmission between photoreceptors and bipolar cells, and progressive photoreceptor degeneration (Weber et al., 2002), since RS1 is critical in stabilizing the synaptic connections during development (Takada et al., 2004; Vijayasarathy et al., 2006, 2008). Retinoschisin interacts with both Ca_v_1.3 and Ca_v_1.4 (Shi et al., 2009, 2017). While LTCCs are critical for RS1 secretion, RS1 augments LTCCs (Ko et al., 2008; Shi et al., 2009, 2017). Thus, the decreased density of synaptic ribbons in the OPL (Figure 6) as well as decreased ERG b-wave in Ca_v_1.3^−/−^ mice might be in part due to the decreased RS1.

In a previous report, with 7-min light pulses, the light peak (LP) of ERGs recorded from Ca_v_1.3^−/−^ is reduced compared to the WT littermates (Wu et al., 2007). The LP of the ERG is caused by a depolarization of the basolateral plasma membrane of the retinal pigment epithelium (RPE). Since Ca_v_1.3 is also expressed in the RPE (Rosenthal et al., 2006), the decreased LP observed in Ca_v_1.3^−/−^ mice further provides evidence that Ca_v_1.3 contributes to retinal light responses. However, contradicting previous reports that Ca_v_1.3^−/−^ mice only have mild decreases in ERG a- and b-waves compared to the WT littermates (Wu et al., 2007; Busquet et al., 2010), we found that the Ca_v_1.3^−/−^ mice had significantly lower ERG a-, b-waves, and OPs compared to the WT littermates and the WT purchased from the vendor. One possible explanation is the recording procedure or instrumentation differences. But in their morphological study, the OPL of the Ca_v_1.3^−/−^ retina has more clusters of puncta or patches when labeled with the synaptic marker Ribeye, differing from the horseshoe-like appearance in the WT retina (Busquet et al., 2010). This observation is similar to our immunostaining with the synaptic ribbon marker Ribeye and Ca_v_1.4 that the ribbon synapse density at the OPL of the Ca_v_1.3^−/−^ mouse retina was decreased compared to the WT (Figure 6, Table 3). Our morphological study showing decreased ribbon synapses in the OPL of Ca_v_1.3^−/−^ retinas echoes the decreased ERG responses recorded from Ca_v_1.3^−/−^ mice. Hence, these results confirm the functional importance of Ca_v_1.3 in retinal physiology.

One major functional role of LTCCs is to govern the tonic neurotransmitter release from the ribbon synapses of photoreceptors and bipolar cells (von Gersdorff et al., 1996; Barnes and Kelly, 2002; Hull et al., 2006b). Both Ca_v_1.3 and Ca_v_1.4 are expressed in the synaptic terminals of photoreceptors. While Ca_v_1.3 is present from the inner segments to synaptic terminals of photoreceptors (Firth et al., 2001; Xu et al., 2002; Morgans et al., 2005; Hull et al., 2006b; Cristofanilli et al., 2007; Ko et al., 2007), Ca_v_1.4 is strongly expressed at the ribbon synapses (Morgans, 2001; Morgans et al., 2005; Liu et al., 2013). Ca_v_1.4 clearly plays a pivotal role in the maintenance of structure and function of the ribbon synapses in the OPL during development, since its deletion causes ribbon synapses to stay in an immature state (Liu et al., 2013). Two major biophysical characteristics of Ca_v_1.4 that differ from other LTCCs are the absence of calcium-dependent inactivation and the slow voltage-dependent inactivation, which make Ca_v_1.4 ideally suited for the tonic calcium influx at the photoreceptor synaptic terminal for neurotransmitter release in the dark (Koschak et al., 2003; McRory et al., 2004). Mutations of *cacna1f*, the gene encoding Ca_v_1.4, cause CSNB2 (Bech-Hansen et al., 1998; Liu et al., 2013). One would expect a total loss of ERG post-photoreceptor components from CSNB2 patients. However, these patients still have a small residual b-wave with slower kinetics (Bradshaw et al., 2004), indicating that there could be other LTCCs present at the photoreceptor-bipolar cell synapses enabling the inner retina to still respond to light signals. Our results showed the density of photoreceptor ribbon synapses decreased in the OPL of Ca_v_1.3^−/−^ retina, which supports the notion that Ca_v_1.3 also contributes to synaptic transmission in photoreceptors and other inner retinal neurons. In cochlea hair cells, Ca_v_1.3 is required for the maintenance of ribbon synapses, and calcium influx through Ca_v_1.3 fine tunes the size of synaptic ribbons during development (Sheets et al., 2012; Joiner and Lee, 2015). This phenomena has also been observed in pinealocyte synaptic ribbons (Sheets et al., 2012) and might be present in other cell types. Our study sheds light that Ca_v_1.3 may also contribute to the maintenance of photoreceptor ribbon synapses.

Although there is evidence showing the presence of Ca_v_1.4 in bipolar cell synapses (Morgans, 2001), we only detected Ca_v_1.4 in the OPL, not IPL, which is consistent with another study (Lee et al., 2015). Our results suggest that Ca_v_1.3 along with Ca_v_1.2, but not Ca_v_1.4, are the major LTCCs in bipolar cells. Compared to Ca_v_1.2, Ca_v_1.3 activates at a more negative voltage and inactivates more slowly during depolarization. Ca_v_1.3 is less sensitive to dihydropyridine inhibition and calcium-induced inactivation than Ca_v_1.2 (Platzer et al., 2000; Xu and Lipscombe, 2001). These biophysical properties make Ca_v_1.3 more suitable to trigger neurotransmitter release in bipolar cells and sustain their depolarization. Therefore, deletion of Ca_v_1.3 dampens the neuro-signal relay in bipolar cells leading to decreased ERG b-waves.

Another aspect of Ca_v_1.3 function is its role in short-term retina adaptation to external stimulation. Activation of glutamate receptors causes a rapid internalization of Ca_v_1.3 in cultured amacrine and ganglion neurons (Mizuno et al., 2010), suggesting that Ca_v_1.3 is highly responsive to changes in light stimulation, and such plasticity of Ca_v_1.3 may serve as an acute adaptation to protect the inner retinal circuitry against glutamate excitotoxicity (Mizuno et al., 2010). Besides the short-term adaptation that the retina possesses in response to light or dark stimulation, the retina is able to undergo longer term adaptation that can last for hours to days, which includes regulation by the intrinsic retinal circadian oscillators (Green and Besharse, 2004). Interestingly, the retinal light responses measured by ERG are under circadian control (Lu et al., 1995; Manglapus et al., 1998; McGoogan and Cassone, 1999; Cameron et al., 2008; Cameron and Lucas, 2009). While the circadian rhythm of ERG a-waves can be explained by the circadian regulation of cGMP-gated cation channels (Ko et al., 2001, 2003, 2004), the rhythmic changes in ERG b-waves have not yet been clearly defined, in which the circadian rhythm of Ca_v_1.3 (Ko et al., 2007, 2013; Ko M. L. et al., 2009; Huang et al., 2013; Lin et al., 2015) might partially explain the circadian rhythmicity of ERG b-waves. In summary, combining the morphological and physiological data, Ca_v_1.3 contributes to synaptic transmission and inner retinal light responses. The role of Ca_v_1.3 in retinal physiology and function is more prominent than previously reported.

## Author contributions

LS and GK designed the experiment. LS and JC, performed the experiment. LS, JC, FY, and GK analyzed the data. LS, MK, and GK wrote the manuscript. LS, JC, FY, MK, and GK edited the manuscript.

### Conflict of interest statement

The authors declare that the research was conducted in the absence of any commercial or financial relationships that could be construed as a potential conflict of interest.

## Supplementary material

Supplementary Figure 1 Statistical analyses of synaptic structures at OPL (the Table 3 data plotted). Each datum point represents the average from a single mouse retina. *N* = 5 (mice) for each group. ^*^*p* < 0.05.
