# Supplementary figures and images for: The Contribution of L-Type Cav1.3 Channels to Retinal Light Responses
Source: Front Mol Neurosci. 2017 Dec 5;10:394. doi: 10.3389/fnmol.2017.00394 (PMC5723326; doi:10.3389/fnmol.2017.00394)

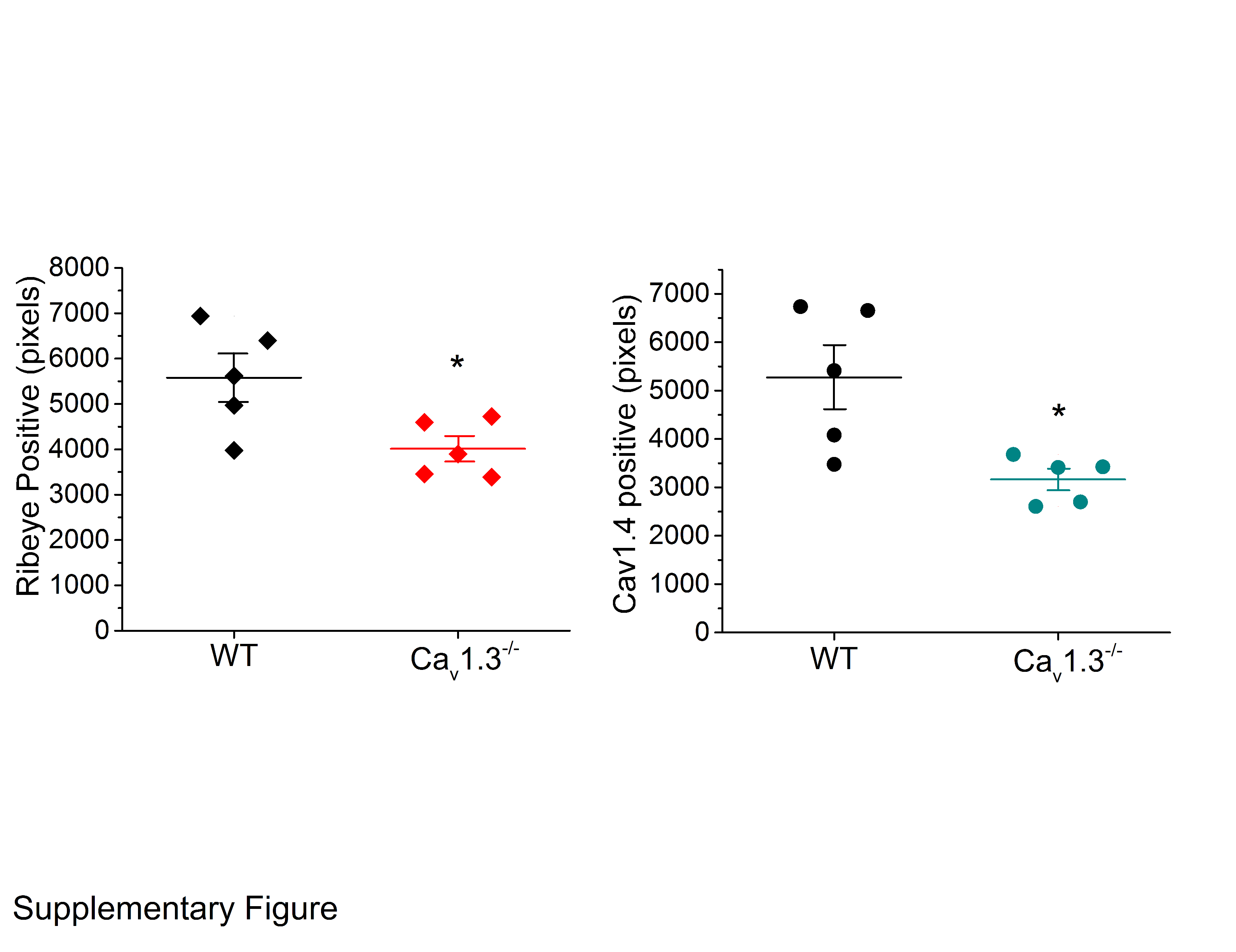

Supplement: Supplementary Figure 1 — Statistical analyses of synaptic structures at OPL (the Table 3 data plotted). Each datum point represents the average from a single mouse retina. N = 5 (mice) for each group. *p < 0.05. [file Image1.TIF]
